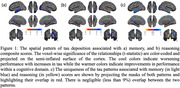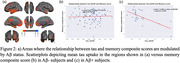# Differential Pattern of Tau Deposition is Associated with Each Cognitive Score in Cognitively Unimpaired Elders

**DOI:** 10.1002/alz.090023

**Published:** 2025-01-03

**Authors:** Siddharth Nayak, Seyed Hani Hojjati, Sindy Ozoria, Peter Chernek, Jenseric Calimag, Bardiya Ghaderi, Qolamreza R Razlighi

**Affiliations:** ^1^ Weill Cornell Medicine, New York, NY USA

## Abstract

**Background:**

Different subtypes of Alzheimer’s Disease (AD) are shown to have differential patterns of tau deposition on the cerebral cortex. However, for cognitively unimpaired elders the spatial specificity of tau deposition has not been fully investigated.

**Objective:**

We aim to show that tau deposition in different brain regions is uniquely associated with performance in different cognitive domains. Furthermore, we investigate the influence of amyloid‐β (Aβ) in every tau‐cognition relationship.

**Methods:**

A total of 124 cognitively unimpaired older adults (68.62 ± 5.56 years) underwent ^18^F‐florbetaben (FBB) positron emission tomography (PET) scans to determine Aβ positivity and ^18^F‐MK‐6240 PET scans for measuring in vivo tau accumulation in addition to four cognitive domain assessments, and structural magnetic resonance imaging (MRI). Voxel‐wise multiple regression analysis is used with voxel‐wise tau uptake as the outcome and cognitive scores, Aβ status and their interaction as the predictor of interest controlling for age, sex, and head size.

**Results:**

Memory and reasoning scores were associated with a unique pattern of tau deposition with negligible spatial overlap (less than 9% on Dice), as shown in Fig. 1, suggesting that each cognitive domain is affected by distinct patten of tau accumulation. Furthermore, as seen in Fig. 2, the presence of Aβ significantly modulates the effect of tau on memory in most regions shown in Fig. 1a in a way that only Aβ+ participants show significant tau‐memory relationships while Aβ‐ participants show no relationship. Strikingly, the tau‐cognitive relationship for other domains did not show strong interactions in the cortex. However, reasoning and speed showed a weak interaction with tau in the right entorhinal cortex and left hippocampus, respectively.

**Conclusion:**

Our findings provide evidence for regional specificity of tau deposition in unimpaired elders, as it has been reported in AD patients. In addition, while the presence of Aβ strongly modulates the relationship between tau and memory, this modulation is significantly attenuated or completely missing from other cognitive domains.